# Role of Nitric Oxide in Cardioprotection by Poloxamer 188

**DOI:** 10.3390/cells14131001

**Published:** 2025-06-30

**Authors:** Zhu Li, Matthew B. Barajas, Takuro Oyama, Matthias L. Riess

**Affiliations:** 1 Department of Anesthesiology, Vanderbilt University Medical Center, Nashville, TN 37232, USA; zhu.li@vumc.org (Z.L.); matthew.b.barajas@vumc.org (M.B.B.); taku0619uro12@gmail.com (T.O.); 2 Department of Anesthesiology, TVHS VA Medical Center, Nashville, TN 37212, USA; 3 Department of Pharmacology, Vanderbilt University, Nashville, TN 37232, USA

**Keywords:** calcium, cardiomyocyte, co-culture, co-polymer, cross-talk, endothelium, hypoxia reoxygenation, lactate dehydrogenase, NO, P188

## Abstract

Poloxamer (P) 188 attenuates myocardial ischemia/reperfusion injury through cell membrane stabilization. Cell–cell interactions between endothelial cells (ECs) and cardiomyocytes (CMs) further protect CMs: co-cultures showed that, at an optimal density, ECs protected CMs against hypoxia/reoxygenation (HR) injury. The mechanism of interaction with P188 still requires exploration. We examined if N(ω)-nitro-L-arginine methyl ester (LNAME), a non-specific nitric oxide (NO) synthase inhibitor, abolishes protection in the presence or absence of P188 and/or ECs. We co-cultured mouse coronary artery ECs in an insert atop mouse CMs plated at confluency on the bottom of a well. Normoxic controls remained in complete media while HR groups were exposed to 24 h hypoxia at 0.01% O_2_ in serum- and glucose-free media, followed by 2 h reoxygenation in complete media. P188 (300 μM), LNAME (40 mM), or vehicle were administered upon reoxygenation. ECs at the used lower density did not decrease HR-triggered lactate dehydrogenase release or calcium overload in CMs by themselves. P188 reduced both indicators after HR by 16/18% without and by 22/25% with ECs, respectively. LNAME abrogated CM protection by P188. Neither intervention had an effect under normoxia. Our co-culture data indicates that P188 requires NO, not necessarily of endothelial origin, to elicit CM protection.

## 1. Introduction

Reperfusion is the restoration of blood flow after an ischemic event. Although reperfusion is required to return cells to normal function, this process can cause additional cellular injury resulting from the reintroduction of oxygen (reoxygenation) to previously oxygen-depleted (hypoxic) tissue, known as ischemia/reperfusion (IR) injury, for which there is still no effective treatment. Reperfusion after prolonged hypoxia may cause endothelial dysfunction [1,2], cell membrane depolarization [3,4], intracellular calcium overload [5,6], and apoptotic cell death [7,8,9,10].

Poloxamer (P) 188, a co-polymer-based cell membrane stabilizer, has been suggested to attenuate myocardial IR injury through its ability to protect cell membrane integrity [11,12,13]. P188 is a non-ionic, tri-block co-polymer consisting of a core lipophilic poly-propylene oxide (PPO) block of 30 units flanked by two hydrophilic poly-ethylene oxide (PEO) blocks of 75 units to form a PEO_75_-PPO_30_-PEO_75_ structure of 8.4 kDa [14,15]. These units allow the chain to integrate itself into the plasma membrane of damaged cells [16,17]. P188 is not a pro-drug and requires no alteration or conjugation. In addition, P188 is not metabolized in vivo and is renally excreted [18]. These properties make P188 an appealing candidate to treat pathologies across several tissue types in vitro and in vivo [11,19,20], as well as to facilitate drug delivery and enhance drug penetration [21,22]. In addition, preclinical studies, including ours, have demonstrated P188’s cardioprotective effect in a variety of IR injury models [23,24,25,26], including animals in vivo [12,13]. P188 is thought to reduce membrane leakage [11,16,27] and apoptosis [28] and to improve membrane integrity and mitochondrial function in vivo [13].

Previous co-culture studies found cell–cell interactions between endothelial cells (ECs) and cardiomyocytes (CMs), the two most abundant cardiac cell types, further protected CMs from simulated IR injury [29,30,31]. We now hypothesize that P188 and ECs act in concert to protect CMs, and that nitric oxide (NO) is an essential part of this pathway.

To fulfill the study’s objectives, we examined if N(ω)-nitro-L-arginine methyl ester (LNAME), a non-specific NO synthase (NOS) inhibitor, could attenuate LDH release from and calcium overload in CMs as two markers of cell membrane damage in the presence of P188 when co-cultured with ECs after prolonged HR injury. The current study provides further important evidence on the mechanism of P188 protecting cardiac function following IR.

## 2. Materials and Methods

### 2.1. Cell Culture

Adult mouse CMs (Catalog No. 11041-14) were purchased from Celprogen Inc. (Torrance, CA, USA) and mouse primary coronary artery ECs (Catalog No. C57-6093) were purchased from Cell Biologics (Chicago, IL, USA). These cells originated from adult C57BL/6 mouse cardiac tissue or coronary arteries, respectively. Kept in complete growth media containing 10% fetal bovine serum (FBS), antibiotics, and supplements, the cells were cultured in a cell incubator with a standard humidified culture environment of 21% O_2_, 5% CO_2_, and 74% N_2_ at 37 °C.

**Figure 1:**
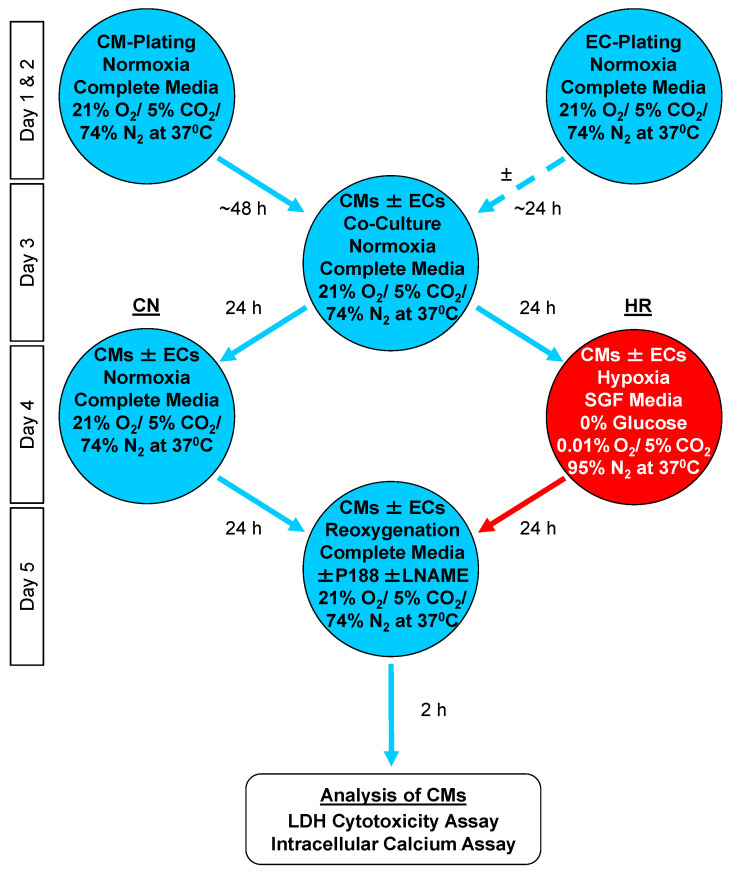
Flow chart of the co-culture model. Cardiomyocytes (CMs) and endothelial cells (ECs) were cultured separately in complete media and under normoxic conditions. Next, either CMs continued to be cultured alone or EC inserts were placed atop the CM wells. This was followed by hypoxia in combination with serum- and glucose-free (SGF) media or by control/normoxia (CN) in complete media before the endpoint assays. HR: hypoxia/reoxygenation; LDH: lactate dehydrogenase.

**Figure 2:**
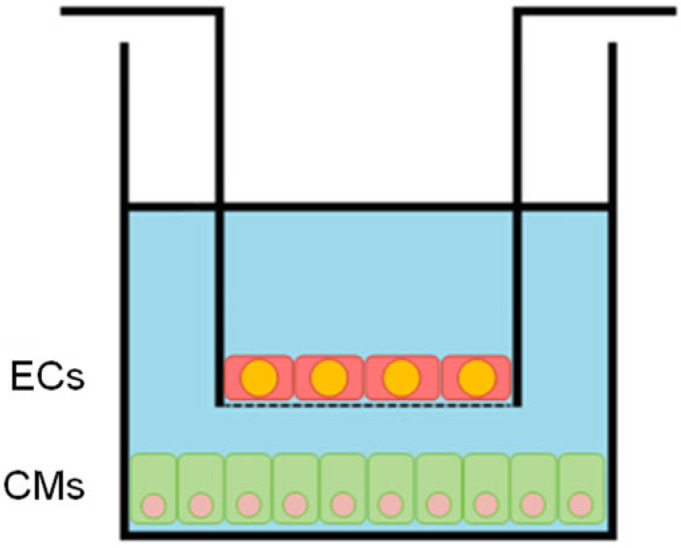
Schematic of the insert with the endothelial cells (ECs) inside the well containing the cardiomyocytes (CMs) on the bottom. The semi-permeable barrier, indicated by a dotted line at the bottom of the EC insert, allows for cell–cell communication without mixing of media. ECs were plated at a lower density of 25,000 cells per insert and CMs at 300,000 per well. Modified from Li et al. [31].

Figure 1 shows the flow chart of the EC-CM co-culture protocol used in this study. On day 1, CMs were plated into 24-well plates. ECs were plated into the inserts (Catalog No. 662640, from Greiner Bio-One, Kremsmünster, Austria) (Figure 2) [31] at 25,000 per insert on day 2; this lower concentration was chosen to not saturate cardioprotection and, thus, provide blunt additional protection of CMs by P188 as previously reported [31]. Co-cultures began on day 3 by inserting the EC-confluent top rack into the well with the CMs after the latter had reached 80% confluency, approximately 48 h after plating. Both cell types were now allowed to co-culture for another 24 h before randomization into control normoxia (CN) or HR groups to simulate IR (day 4 to 5). On day 5, after 24 h of hypoxia and 2 h of reoxygenation, the biological endpoint assays were performed on the CMs.

### 2.2. In Vitro HR Injury

When the cells in the 24-well plates or cell inserts reached confluency, the cell plates for each experiment were randomized to CN or HR groups. IR was simulated by starvation of O_2_ and nutrients and by placing the cells into serum- and glucose-free (SGF) media in a humidified Billups-Rosenthal plexiglass hypoxia chamber (Stemcell Technologies; Vancouver, BC, Canada), flushed with a hypoxic gas mixture (0.01% O_2_, 5% CO_2_, and 95% N_2_), and placed in the cell incubator at 37 °C for 24 h [31]. This insult is stronger than previously used in CMs [24]. CN cells were kept under normal conditions; regular serum- and glucose-containing cell media were refreshed at the same time points as the corresponding HR cells. When the cells were removed from the hypoxic chamber, the 2 h reoxygenation period started. The media were refreshed with regular media, and the experimental plates were returned to the normal culture environment (21% O_2_, 5% CO_2_, 74% N_2_, and 37 °C).

After the 2 h reoxygenation period, cell inserts were removed and CMs or cell culture media from all the groups were transferred from 24-well plates into 96-well plates to perform the biological assays in a 96-well plate reader. After the transfer of the cells, 100 µL trypsin and 500 µL of the media were used for each well; cells were quickly spun at about 80 g for 1 min and were then divided into 96 wells (50 µL per well) to be read in the plate reader. For each control and treatment experiment, multiple replicates were plated.

### 2.3. LDH Cytotoxicity Assay

We used the LDH Cytotoxicity Assay Kit (Pierce Biotechnology, Rockford, IL, USA) [25,31] to measure the intracellular enzyme LDH released from damaged cells into the culture media; this assay is widely used as an indicator of cell membrane damage and cytotoxicity.

At the end of the 2-h reoxygenation period, 50 µL of the media from the CM portion of each well was transferred to a corresponding well of a new 96-well plate and mixed with 50 µL of the prepared LDH reaction mixture. Following 30 min incubation whilst protected from light and at room temperature, reactions were terminated by addition of 50 µL of a stop solution, halting the reduction of the tetrazolium salt to the formazan product. Absorbance was measured at 490 nm in a plate reader.

### 2.4. Fluo-4 Direct Calcium Assay

The Fluo-4 Direct Calcium Assay Kit (Catalog No. F10471; Molecular Probes, Inc., Eugene, OR, USA) was used to measure intracellular calcium [24]. The advanced formulation of this kit allows the assay to be run as a simple addition without removing the cell culture media. After the cells were transferred into 96-well plates, 50 µL of the prepared 2× Fluo-4 direct working solution was added into the wells containing cells and 50 µL of the regular culture media. After incubating at 37 °C for about 30–60 min, the plates were read in the 96-well plate reader at an excitation of 494 nm and emission of 576 nm.

### 2.5. Treatment with P188 and LNAME

Since timing of P188 administration is critical as it is most effective when given just before or during reperfusion [13], P188 (300 µM) and LNAME (40 mM) were given at the clinically important time of reoxygenation. Both drugs were applied to both ECs and CMs during medium refreshing. We chose P188’s concentration based on studies that have investigated the cellular protective potential of P188. While in mouse muscle myoblast cultures (~80% confluent) injured by hypo-osmotic stress and isotonic recovery, 14 µM P188 was protective and 150 µM P188 fully restored dystrophic mouse myocyte stretch compliance [32], results from ongoing studies and our previously published data [24,25] led us to choose 300 µM to further study the interaction with ECs and NOS inhibition. All drugs were dissolved in regular cell media to achieve the desired concentrations. At these concentrations, they are completely soluble.

### 2.6. Statistics

All experiments were replicated in triplicate at minimum. All data within one experimental group were normalized to %CN in the absence of ECs, P188, and LNAME. Data were tested for normal distribution. Statistical differences among groups were analyzed by a One-Way Analysis of Variance followed by Student–Newman–Keuls post hoc comparisons for normally distributed data. To confirm and complement these multiple comparisons, multiple linear regression analyses were also conducted, with the presence of LNAME, ECs, P188, and HR as independent variables. A *p*-value < 0.05 (two-tailed) was considered to be statistically significant. All data are shown as the mean ± standard error of the mean. Significance symbols are * vs. Con, † vs. EC, and # vs. CN. Sigma Stat 3.5 (Systat Software, San Jose, CA, USA) was used for the statistical analyses.

## 3. Results

### 3.1. LDH Release

**Figure 3:**
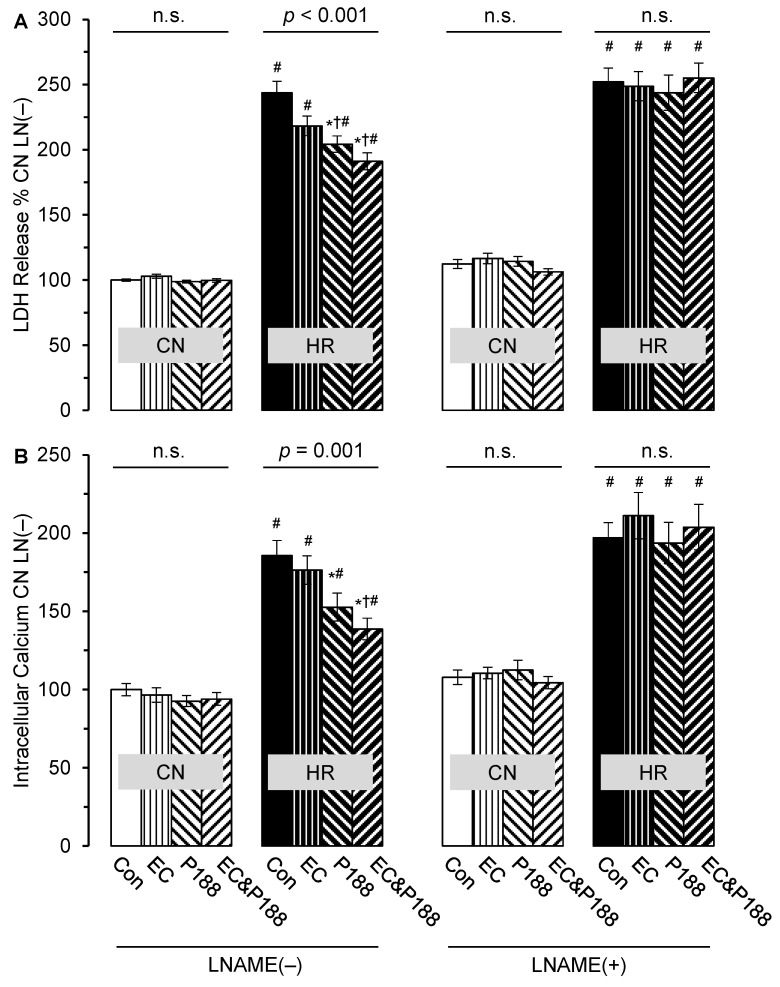
Release of lactate dehydrogenase (LDH; panel (**A**)) and intracellular calcium (panel (**B**)) 2 h after reoxygenation following hypoxia (HR) vs. control/normoxia conditions (CN) in the absence (−) or presence (+) of the nitric oxide synthase inhibitor N(ω)-nitro-L-arginine methyl ester (LNAME; 40 mM) from isolated cardiomyocytes. Experiments were conducted in the absence (Con) or presence of co-cultured endothelial cells (EC; 25,000 per insert), Poloxamer (P) 188 (300 µM), or both. All data are shown as mean ± standard error of the mean normalized to 100% of CN conditions in the absence of ECs, P188, and LNAME; n = 5 to 6 per group, with at least 3 replicates per experiment. Statistics: One-Way Analysis of Variance followed by Student–Newman–Keuls post hoc comparisons, *p* < 0.05 (two-tailed), with * vs. Con, † vs. EC, and # vs. CN; n.s. = not significant.

In the absence of LNAME, HR led to a ~2.5-fold increase in LDH release from CMs compared with CN (Figure 3A). This increase by HR was significantly attenuated by 16% in the presence of P188 (*p* = 0.002) and by 22% in the presence of ECs and P188 (*p* < 0.001), whereas ECs alone had no statistically significant positive effect (*p* = 0.135); LDH in ECs and P188 was also not significantly lower than in P188 alone (*p* = 0.237).

Under CN conditions, neither the presence of ECs nor P188 nor their combination had any significant effect on LDH release (*p* = 0.393).

When LNAME was added, it had no effect on LDH release under CN conditions (*p* = 0.381) and it had no effect on the increase in LDH release by HR, but it completely abolished the observed attenuation of the HR-induced increase in LDH release by P188 as well as ECs and P188 (no difference among the HR groups; *p* = 0.923).

A multiple linear regression analysis across all groups with LDH release as the dependent variable and HR, LNAME, ECs, and P188 as independent variables confirmed the above findings: although HR (*p* < 0.001), LNAME (*p* < 0.001), and P188 (*p* = 0.006) significantly affected LDH release, the presence of ECs did not (*p* = 0.293). A further analysis in the HR LNAME(−) group only, however, showed LDH release to significantly depend on the presence of ECs (*p* = 0.018) and P188 (*p* < 0.001), but not on their product (*p* = 0.406), i.e., no significant interaction between ECs and P188 was found.

### 3.2. Intracellular Calcium

Similar to the release of LDH, HR caused a ~2-fold increase in intracellular calcium in CMs compared with CN (Figure 3B). This increase was also attenuated by 18% by P188 alone (*p* = 0.026) and by 25% by its combination with ECs (*p* = 0.002), whereas ECs alone had no positive effect on calcium overload (*p* = 0.451). Calcium in the presence of ECs and P188 was also not significantly lower than with P188 alone (*p* = 0.271).

In contrast to HR, neither the presence of ECs nor P188 nor their combination affected intracellular calcium under CN conditions (*p* = 0.599).

As seen with LDH release, LNAME completely abolished the observed attenuation by P188 with and without ECs on intracellular calcium overload by HR (no difference among the HR groups; *p* = 0.796), while it had no effect under CN conditions (*p* = 0.656).

A multiple linear regression analysis across all groups with intracellular calcium as the dependent variable and HR, LNAME, ECs, and P188 as independent variables also confirmed the above findings: although HR (*p* < 0.001), LNAME (*p* < 0.001), and P188 (*p* = 0.007) significantly affected intracellular calcium, the presence of ECs did not (*p* = 0.740). In contrast to LDH, a further analysis solely on the HR LNAME(−) group showed intracellular calcium to significantly depend on P188 (*p* < 0.001) only, but not the presence of ECs (*p* = 0.188) nor their product (*p* = 0.406), i.e., there was no significant interaction between ECs and P188 in this context.

## 4. Discussion

P188 has been investigated as an intervention for pathologies across several tissue types and has been studied in vivo and in vitro, as well as in clinical trials [33,34]. It has several additional biological applications such as facilitating drug delivery [21], and enhancing drug penetration [22]. All these properties are directly linked to its affinity to amphiphilic phospholipid membranes [35].

Bartos et al. [13] showed that, when given directly into the circumflex coronary artery upon reperfusion after myocardial ischemia for 45 min by balloon occlusion, P188 largely improved outcomes in an in vivo porcine model: P188 improved the infarct size, troponin leakage, mitochondrial yield, respiratory control index, and calcium retention capacity of the cardiac mitochondria compared with the control. These positive findings were in stark contrast to the neutral results of the previously conducted CORE trial, where P188, given intravenously and at a later time point, failed to demonstrate any beneficial effect in patients [33]. Bartos et al. found an explanation for this discrepancy by also administering P188 intravenously and later, which did not lead to any protection in any of the assessed outcomes in their study either [13].

We could corroborate P188’s cardioprotective effects in isolated CMs [24] and in a rat isolated heart model: P188, given immediately upon reperfusion after 30 min of global ischemia, improved diastolic contracture, developed left ventricular pressure and contractility, and attenuated the infarct size after reperfusion [26]. Furthermore, in our Langendorff model, the non-specific NOS inhibitor LNAME abolished P188-induced cardioprotection completely, and P188 dose-dependently increased the production of NO, as evidenced by NO-specific fluorescence [26]; it was also abolished by LNAME, providing the first evidence that NO might indeed play a crucial role in mediating cardioprotection by P188 in intact organs ex vivo and, thus, possibly in vivo.

In parallel, additional work showed that the tri-block co-polymer P188, as well as further-improved di-block co-polymers [35], also protected isolated coronary ECs against HR injury in a dose-dependent manner [25]. The same was true for P188 administered to brain vascular ECs undergoing HR, compression, or a combination thereof to mimic stroke, concussion, or TBI, respectively [36].

Collaborators of ours used human induced pluripotent stem cells to produce human ECs and isolated contracting CMs. They also demonstrated a dose-dependent increase in NO production and improved cellular function and protection against HR injury by P188 in both cell types, respectively [26], which, in part, corroborated our earlier findings of P188 protecting isolated non-contracting CMs against HR injury in a dose-dependent manner [24,25]. However, Chen et al. [26] separately studied both cell types, ECs and CMs, but not together, as in our co-culture model [31].

The present study was conducted in an attempt to close this knowledge gap by using an established co-culture model of isolated cardiac ECs and CMs [31], in which we administered P188 and/or LNAME to explore intercellular cross-talk and the role of (endothelial) NO in the P188-induced protection of CMs.

To investigate the role of autocrine and/or paracrine cell–cell interactions on cell function and differentiation, cell co-culture models have been used extensively. CMs and ECs represent the two most abundant cell types in the heart. In vivo, IR damages different types of cells in different ways. In the heart, the inflammation and oxidative stress of CMs cause most of the pathological responses to IR, but IR also damages surrounding cells, especially ECs. EC dysfunction aggravates CM injury and, consequently, results in injury expansion, possibly leading to cell death. On the other hand, ECs can also provide cytoprotection for CMs during IR, emphasizing a vital role of EC-CM cross-talk: co-culture studies found that cell–cell interactions between ECs and CMs can further protect CMs from IR injury [29,31,37]. Data from our co-culture model showed that the ability of P188 to promote cardioprotection depended on an appropriate EC plating density. At a higher density of 100,000 per well, ECs appeared to saturate cardioprotection by themselves and blunt any additional protection of CMs by P188; in contrast, at a lower density of only 25,000 per well, they had no intrinsic cardioprotective effect and allowed P188 to reduce LDH release from CMs following HR [31]. Thus, the lower density of 25,000 per well was used in the present study.

Our data on LDH release and calcium overload as markers of cell membrane damage by HR in the present study reveal some ambiguity. In the absence of LNAME, the results do not suggest an interaction of P188 with ECs at the chosen density: there was only a statistically significant difference between P188 and the EC and P188 groups vs. Con and vs. ECs alone, but there was no significant difference between the EC and P188 vs. P188 group alone. Therefore, P188 does not seem to require the presence of ECs, and ECs at the chosen lower plating density did not significantly enhance P188’s ability for cellular protection. On the other hand, as the non-specific NOS inhibitor LNAME completely abolished any protection by P188 or ECs and P188 in our co-culture—as it did in the isolated, beating heart preparation [26]—this reconfirmed that NOS, not necessarily endothelial NOS, is indeed required for P188 to exert its protective effects on CMs. It also corroborates our findings in both the Langendorff model and in human isolated ECs [26], where P188 dose-dependently increased NO production.

Limitations and outlook: Naturally, the results of our study need to be interpreted within their given constraints. (a) Isolated cells, even in an elaborate co-culture model such as ours, cannot replace the intricate interplay among cells and between cells and other factors present in whole organs ex vivo, not to mention in vivo. As laid out above, though, they can complement these models to further explore the mechanisms of cellular protection and cross-talk. (b) We used only one set of conditions (cell types, HR durations, cell density, distance between ECs and CMs, drug concentrations, and outcome measures, to name only a few) that cannot necessarily be generalized to other conditions and might not perfectly reflect the situation in vivo. However, they were the optimal result of careful titrations and numerous preliminary experiments. (c) As both CMs and ECs can release LDH, we cannot distinguish between the two. The release of LDH, however, is generally more prominent from CMs than from ECs due to their higher LDH content and increased vulnerability to HR. Had the addition of ECs contributed significantly, the measured LDH would have been higher in the two groups with ECs present than in those without, which was not the case. (d) While focusing on NO, we did not study reactive oxygen species or other potential paracrine factors that might also play an important role in this context. (e) The complex study design with 2^4^ groups, resulting in multiple comparisons, may have led to a type 2 error despite many replicates and the number of independent experiments, specifically among the HR LN(−) groups, where the P188 and ECs group was not statistically different from P188 alone. (f) Most notably, we only employed LNAME and no other NOS inhibitors that would have been more specific for neuronal or inducible NOS. (g) Future experiments will need to explore the downstream targets of NOS in CMs to further elucidate how P188 elicits cardioprotection through the NO signaling pathway [38]. (h) This poses the question of whether P188 still works under conditions of reduced NO production [39] such as hypertension, diabetes, artherosclerosis, kidney disease, and old age, topics of high interest for possible future clinically oriented explorations.

## 5. Conclusions

These and previous data from our co-culture model of CMs with ECs indicate that (a) oxidative injury, caused by reoxygenation after prolonged hypoxia and assessed by cellular LDH release and intracellular calcium measurement, can be attenuated by the tri-block co-polymer P188 administered upon reoxygenation; (b) alternatively, ECs can protect CMs, if present at a high enough density; (c) P188 does not necessarily require the presence of ECs to protect CMs; and (d) P188 requires NO signaling for the protection of CMs, as shown by the complete abrogation of its protective effect by the non-specific NOS inhibitor LNAME. The exact role of the NO signaling pathway and its clinical implications remain to be further investigated in the future.

## Data Availability

All data are available upon reasonable request and in accordance with all funding guidelines.

## Abbreviations

The following abbreviations are used in this manuscript:

CM(s)	Cardiomyocyte(s)
CN	Control normoxia
EC(s)	Endothelial cell(s)
HR	Hypoxia reoxygenation
IR	Ischemia reperfusion
LDH	Lactate dehydrogenase
LNAME	N(ω)-nitro-L-arginine methyl ester
NO	Nitric oxide
NOS	Nitric oxide synthase
P	Poloxamer
PEO	Poly-propylene oxide
PPO	Poly-ethylene oxide
SGF	Serum- and glucose-free
